# Knowledge and practices of private pharmacy auxiliaries on malaria in Abidjan, Côte d’Ivoire

**DOI:** 10.1186/s12936-023-04751-8

**Published:** 2023-11-02

**Authors:** Valérie A. Bedia-Tanoh, Étienne K. Angora, Sebastien A. J. Miezan, Estelle D. M. Koné-Bravo, Abibatou Konaté-Touré, Henriette Bosson-Vanga, Fulgence K. Kassi, Pulchérie C. M. Kiki-Barro, Vincent Djohan, Hervé E. I. Menan, William Yavo

**Affiliations:** 1 Pharmaceutical and Biological Sciences Training and Research Unit, Parasitology and Mycology Department, UFHB, PO Box V34, Abidjan, Côte d’Ivoire; 2 Research and Control Malaria Centre, National Public Health Institute, PO Box V 47, Abidjan, Côte d’Ivoire; 3 Diagnostic and Research Centre on AIDS and other Infectious Diseases (CeDReS), CHU Treichville, PO Box V 13, Abidjan 01, Côte d’Ivoire

**Keywords:** Pharmacy auxiliaries, Practices, Knowledge, Malaria

## Abstract

**Background:**

The emergence of resistance to artemisinin derivatives in Southeast Asia constitutes a serious threat for other malaria endemic areas, particularly in Côte d’Ivoire. To delay this resistance, the application of the control measures recommended by the National Malaria Control Programme (NMCP) for a correct management, in the private pharmacies, is a necessity. The purpose of this study was, therefore, to assess the level of knowledge and practices of private pharmacy auxiliary in Abidjan about the management of malaria.

**Methods:**

A descriptive cross-sectional study was conducted from April to November 2015. It included auxiliaries of private pharmacies in Abidjan. Data collection material was a structured an open pretested questionnaire. Data analysis was carried out using Package for Social Science (SPSS) software version 21.1. Chi square test was used to compare proportions for a significance threshold of 0.05 for the p value.

**Results:**

A total, 447 auxiliaries from 163 private pharmacies were interviewed. It was noted that the auxiliaries had a good knowledge of clinical signs of uncomplicated malaria (99.1%), biological examinations (54.6% for the thick film and 40.7% for rapid diagnostic tests (RDTs) and anti-malarial drugs (99.3% for artemether + lumefantrine, AL). The strategies of vector control (long-lasting insecticide-treated mosquito nets (LLITNs, Repellent ointments, cleaning gutters, elimination of larvae breeding site and intermittent preventive treatment with sulfadoxine–pyrimethamine (IPTp-SP) in pregnant women were also known by the auxiliaries, respectively 99.8% and 77.4%. However, the malaria pathogen (25.1%) and the NMCP recommendations (e.g. use of AL or AS + AQ as first-line treatment for uncomplicated malaria and IPTp-SP in pregnant women) were not well known by the auxiliaries (28.2% and 26.9% for uncomplicated and severe malaria). Concerning the practices of the auxiliaries, 91.1% offered anti-malarial drugs to patients without a prescription and 47.3% mentioned incorrect dosages. The combination artemether + lumefantrine was the most recommended (91.3%). The delivery of anti-malarial drugs was rarely accompanied by advice on malaria prevention, neither was it carried out on the result of an RDT.

**Conclusion:**

The epidemiology and the NMCP recommendations for the diagnostic and therapeutic management of malaria, are not well known to auxiliaries, which may have implications for their practices. These results show the need to sensitize and train private pharmacy auxiliaries, and also to involve them in NMCP activities.

## Background

Malaria is a disease caused by a *Protozoa* of the genus *Plasmodium* and is transmitted through the bite of infested female *Anopheles*. Approximately 247 million cases of malaria were reported in 2021, 95% of which are in the African region of the World Health Organization (WHO), with an annual death count of 619,000 [1].

In Côte d’Ivoire, malaria remains a major public health problem due to its high frequency, severity, and important socioeconomic consequences. This parasitic infection represents the main cause of morbidity and accounts for 33% of the reasons for health facility consultation, with an incidence rate of 155 per 1000 in the general population [2] and 281.8 per 1000 in children under 5 years of age [3].

In line with WHO recommendations, most sub-Saharan African countries have shifted to the use of artemisinin-based combination therapy (ACT) as first- and/or second-line malaria treatment since 2005 [4]. Several artemisinin-based combinations are currently used for the first-line treatment of uncomplicated malaria in Côte d’Ivoire, including artemether + lumefantrine (AL), artesunate + amodiaquine (AS + AQ), dihydroartemisinin + piperaquine (DHAP), and artesunate + pyronaridine (ASPY) [5]. The adoption of new treatment policies is confronting several challenges related to inadequate diagnosis and the inappropriate use of drugs due to poor distribution practices, the inadequate labelling of packages, and inappropriate instructions provided to clients on their use [6–8]. Thus, to ensure the effective use of new treatment guidelines and recommendations, both the public and private sectors must be involved. However, private-sector involvement in actions and campaigns organized by the National Malaria Control Programme (NMCP) (known locally as the Programme National de Lutte contre le Paludisme, PNLP) is almost non-existent [9]. When appropriate guidelines are not followed, this can have an impact on the risk of developing drug resistance [10]. In a country where, approximately 40% of patients directly attend private pharmacies without first visiting a health facility [11], the failure to include the private sector, and pharmacies in particular, in the national malaria control strategy can be highly detrimental to efforts to eliminate malaria. The reasons why people prefer pharmacies to public establishments are accessibility, longer opening hours and faster service [8, 12, 13]. Different authors have demonstrated this problem of adequacy and adherence between a national care policy and real practice of health stakeholders in other African countries, such as Nigeria, Mali, and Ghana [14–16].

A study conducted in Malawi, reported that most of the health professionals know about ACT and treatment guidelines for malaria [10]. However, another study showed that pharmaceutical staff working in private pharmacies did not apply malaria management guidelines, which contributed to their lack of knowledge and skills on how to dispense drugs correctly [17]. Furthermore, Hussain et al. were found that the overall knowledge and training of dispensers working at community pharmacies in Pakistan, is inadequate [6]. In the private pharmacies, the auxiliaries are the first people that patients see in the management of their health problems. In 2006, the results of a study conducted in Abidjan indicated a low level of knowledge among these auxiliaries about malaria [18]. So, it is necessary to refresh data. An inventory of the malaria knowledge and practices of private pharmacy assistants as well as the identification of factors associated with these practices could allow the development of actions to strengthen their capacities in the correct management of malaria and thus protect the artemisinin-based combination against the rapid development of *Plasmodium falciparum* resistance. Therefore, the aim of this study was to evaluate the level of knowledge and practices of pharmacy assistants in private pharmacies in Abidjan concerning malaria.

## Methods

### Study area

This was a cross-sectional survey conducted from April to November 2015, in a private pharmacy in Abidjan, the economic capital of Côte d’Ivoire (Ivory Coast). The list of private pharmacies in Abidjan was obtained from the National Order of Pharmacists of Côte d’Ivoire. This list included all the pharmacies in the District of Abidjan (486 pharmacies grouped into 8 sections) (Table 1).

**Table 1:** Distribution of pharmacies by section

Section	Sub-section	Total number of pharmacies distributed by sub-section	Number of pharmacies to be visited by sub-section
Abobo	Abobo	54	18
	Anyama	6	2
Adjamé	Adjamé	31	10
	Attécoubé	14	5
	Williamsville	6	2
Cocody	Riviera	36	12
	Akouédo–Faya–Abatta–Cité SIR	9	3
	Bingerville	5	2
	Cocody	17	6
	II Plateaux/Angré	48	16
Koumassi	Koumassi	37	12
	Port-Bouet–Adjouffou–Jean Folly–Gonzague	7	2
	Port–Bouet	12	4
	Vridi	5	2
Marcory	Marcory	38	13
Plateau	Plateau	21	7
Treichville	Treichville	33	11
Yopougon	Yopougon	107	36
Total		486	163

### Data collection

The district of Abidjan includes 486 pharmacies (grouped into 8 sections) distributed in the 13 communes (Table 1). Thus, to obtain a representative sample of the auxiliaries of private pharmacies in Abidjan, the stratified sampling method has been chosen. The communes of Abidjan represented the strata. The number of pharmacies in this study sample was defined according to the diversity of pharmacies in each community. The community of Bingerville had the smallest number of dispensaries (5); thus, the equiprobability was 1/5. One-third (1/3) of this population was sampled to ensure a minimum of two pharmacies per community. This factor (1/3) was then assigned to the number of pharmacies in each section. Thus, the number of pharmacies to be drawn in each section was distributed in Table 1. In the pharmacy, before any interview with the auxiliaries, a survey authorization issued by the National Union of Private Pharmacists of Côte d’Ivoire (SNPP-CI) was presented to the pharmacist in charge to obtain their agreement to administer the survey. In case of refusal or prolonged absence of the incumbent pharmacist, another selected was made of the remaining pharmacies in the community to ensure the quota of pharmacies to be visited was met. The auxiliaries from private pharmacies in Abidjan were randomly samples. Auxiliaries with at least 3 years of experience in their role with or without specialized training were included after obtaining free and informed consent. All pharmacy assistants, who were selected at random and met the selection criteria, were interviewed.

Data collection material was structured an open pretested questionnaire. this questionnaire was administered to the different auxiliaries of the selected pharmacies to collect data on the level of knowledge and the different practices in the management of malaria. The data collected concerned socio-demographic data (gender, age, level of education, name of training institution, number of years of practice), knowledge of malaria (epidemiology, clinical signs, biological tests, treatment and prevention) and the auxiliaries’s practices in the management of malaria in the pharmacy. The participants in this study were informed of the objectives of the study and regarding confidentiality on the answers provided during the interview, anonymity, and their right to withdraw at any time. The survey was conducted by two teams of sixth-year pharmacy students. The interviewers’ responsibilities were to survey the auxiliary and accurately transcript their responses in the survey form. Some auxiliaries completed the questionnaire themselves. Once in the pharmacy, all of them that accepted to participate in the study, were surveyed.

### Ethical considerations

The survey was based solely on questioning, which posed no danger to the interviewees. On the contrary, it enabled a fruitful exchange of information between the interviewer and the auxiliaries. Participation in the survey was free and without constraint, and all interviewees could voluntarily stop their participation at any time during the study.

### Statistical analysis

The data collected were analysed using the Statistical Package for Social Science (SPSS) version 21.1.0 software. Parameters such as proportions, mean, standard deviation, minimum and maximum were used to describe the study population. Percentages were calculated in relation to the total number of respondents per question. Responses to open ended questions were grouped into common themes. The relationship between two variables was tested with Pearson’s chi-square test or Fisher’s exact test with a 5% risk of error.

## Results

### Sociodemographic characteristics of study participants

A total of 447 auxiliaries from 163 pharmacies (163/486) were interviewed. Most of the auxiliaries were women (83.2% with sex ratio = 0.2). The average age was 34.31 years (standard deviation = 0.62 years) with extremes of 20 and 59 years. They had an average of 7 years of experience. The majority had high school education (91%). In one pharmacy, about one in four auxiliaries learned the trade on the job (Table 2).

**Table 2:** Socio-demographic characteristics of study participants

Characteristics	Number (%)
Gender	
Female	372 (83.2%)
Male	75 (16.8%)
Marital status	
Single	235 (52.6%)
Married	92 (20.6%)
Concubinage	118 (26.4%)
Widowed	2 (0.4%)
Level of education	
Primary	3 (0.7%)
Secondary	407 (91%)
Supérior	37 (8.3%)
Licensed auxiliaries	
Yes	343 (76.7%)
No	104 (23.3%)

### Knowledge of malaria

Only 25.1% of the auxiliaries knew that *Plasmodium* is the pathogen responsible for malaria, but the main mode of transmission of malaria was known by the majority of auxiliaries (93.9%). Garbage and stagnant water were the mosquito breeding sites most frequently mentioned by the workers, by 88.1% and 86.6%, respectively (Table 3). The clinical signs of uncomplicated malaria were well-known to the participants. Almost all the auxiliaries (99.1%) mentioned fever, followed by headache (93.5%) and body aches (89.9%). Concerning the biological diagnosis of malaria, the first test mentioned by the auxiliaries was the thick drop (54.6%), followed by the Rapid Diagnostic Test (RDT) (40.7%) (Table 3). Artemisinin-based combinations were the most known drugs: artemether + lumefantrine by 99.3% and artesunate + amodiaquine by 72.3% of respondents. Less than half of the auxiliaries (45.9%) knew about the National Malaria Control Programme (NMCP) in Côte d’Ivoire. Only 28.2% and 26.9% of the auxiliaries were aware of the management guidelines for simple and severe malaria, respectively (Table 3). A statically significant relationship between knowledge of anti-malarials and having received basic paraprofessional training (p = 0.0001) was observed. However, untrained auxiliaries (36.5%) were more aware of malaria recommendations (Table 4). Visits by medical sales representatives (94.2%) and training courses organized by laboratories (32%) were the main sources of information on malaria for auxiliaries (Table 3).

**Table 3:** Distribution of auxiliaries according to their knowledge and practice on malaria

Variables	Number (%)
Knowledge	
Pathogen	
Good response	112 (25.1)
Poor response	293 (65.5)
Don’t know	42 (9.4)
Mode of transmission	
Oil consumption	1 (0.2)
Heavy work	2 (0.5)
Exposure to the sun	4 (0.9)
Poor hygiene	20 (4.5)
Bite of the female anopheles	420 (93.9)
Major clinical signs	
Fever	443 (99.1)
Headache	418 (93.5)
Body aches	402 (89.9)
Biological diagnosis	
Thick drop	244 (54.6)
Rapid Diagnostic Test (RDT)	182 (40.7)
Blood cell count	52 (16.1)
Thin smear	7 (1.6)
Quantititive Buffy Coat (QBC)	2 (0.4)
Widal and Felix	13 (2.9)
Don’t know	55 (12.3)
Known antimalarial drugs	
Artemether + lumefantrine	444 (99.3)
Artesunate + amodiaquine	323 (72.3)
Dihydroartemisinin + piperaquine	212 (47.4)
Quinine	287 (64.2)
Artésunate + sulfamethoxypyrazine + pyrmethamine	11 (2.5)
Sulfadoxine + pyrimethamine	188 (42.1)
Arterolanemaleate + piperaquine	60 (13.4)
Mefloquine	71 (15.9)
Artesunate + mefloquine	14 (3.1)
Chloroquine	8 (1.8)
Neem Kinkeliba Khaya Senegalensis (DIE KOUADIO)	2 (0.4)
Existence of National Malaria Control Programme (NMCP)	
Yes	205 (45.9)
No	242 (54.1)
Knowledge of NMCP guidelines	
Treatment of uncomplicated malaria	
Good	126 (28.2)
Poor	77 (17.2)
Don’t know	244 (54.6)
Treatment of complicated malaria	
Good	120 (26.9)
Poor	40 (8.9)
Don’t Know	287 (64.2)
Strategics of vector control	
long-lasting insecticide treated mosquito nets (LLITNs)	446 (99.8)
Praying of insecticide	367 (82.1)
Cleaning of gutters	335 (74.9)
Fine wire mesh	149 (33.3)
Repellent ointments	280 (62.6)
Vaccination	38 (8.5)
Elimination of larvae breeding sites	317 (70.9)
Practices	
Advice	
Yes	408 (91.3)
No	39 (8.7)
Delivery of drug and prevention advice	
Yes	2 (0.5)
No	447 (99.5)
Auxiliaire performing RDT	
Yes	151 (33.8)
No	296 (66.2)

**Table 4:** Knowledge and practice of auxiliaries by trained and untrained status

Variables	Specialized		P value
	Yes (%)	No (%)	
Knowledge			
Pathogen			
Good response	75 (21.9%)	37 (35.6%)	*0.0181*
Poor response	234 (68.2)	59 (56.7)	
Don’t know	34 (9.9)	85(7.7)	
Antimalariques			
Artemether + lumefantrine	340 (99.1)	104 (100)	*0.0004684*
Artesunate + amodiaquine	249 (72.6)	74 (71.1)	
Dihydroartemisinin + piperaquine	160 (46.6)	52 (50)	
Quinine	205 (59.8)	82 (78.8)	
Artesunate + sulfamethoxypyrazine + pyrimethamine	8 (2.3)	3 (2.9)	
Sulfadoxine + pyrimethamine	141 (41.1)	47 (45.2)	
Arterolane maleate + phosphate de piperaquine	47 (13.7)	13 (12.5)	
Mefloquine	32 (9.3)	39 (37.5)	
Artesunate + mefloquine	5 (1.4)	9 (8.6)	
Chloroquine	2 (0.6)	6 (5.8)	
Neem Kinkeliba Khaya Senegalensis (DIE KOUADIO)	0 (0)	2 (1.9)	
National treatment guidelines for uncomplicated malaria			
Good	88 (25.6)	38 (36.5)	*0.000081*
Poor	49 (14.3)	28 (27)	
Don’t know	206 (60.1)	38 (36.5)	
National treatment guidelines for complicated malaria			
Good	72 (21)	48 (46.2)	*0.0000011*
Poor	30 (8.7)	10 (9.6)	
Don’t know	241 (70.3)	46 (44.2)	
Practices			
Drugs recommended			
Artemether + lumefantrine	307 (89.5)	101 (97.1)	
Artesunate + amodiaquine	51 (14.9)	27 (26)	
Dihydroartemisinin + piperaquine	66 (19.2)	39 (37.5)	
Quinine	16 (4.7)	14 (13.5)	
Arterolane maleate+ phosphate piperaquine	7 (2)	1 (1)	
Sulfadoxine + pyrimethamine	48 (14)	22 (21.1)	
Mefloquine	3 (0.9)	0 (0)	
Artesunate + mefloquine	0 (0)	1 (1)	
Artesunate + sulfamethoxypyrazine + pyrimethamine	3 (0.9)	0 (0)	
Neem + Kinkeliba + Khaya Senegalensis (DIE KOUADIO)	0 (0)	1 (1)	
None	36 (10.5)	3 (2.2)	
Recommended posology			
Correct	1030 (50.1)	423 (60.3)	*0.0000035*
Incorrect	1025 (49.9)	278 (39.7)	
Auxiliaries performing Rapid DiagnosticTest (RDT)			
Yes	126 (36.7)	25 (24)	*0.0164*
No	217 (63.3)	79 (76)	
What to do in case of negative RDT			
Treat for malaria	28 (8.2)	4 (3.8)	*0.0577*
Treat the symptoms then wait 3 days if symptoms persist, do a curative treatment	1 (0.3)	1 (1)	
Treat the symptoms and/or refer to a health center	106 (30.9)	22 (21.1)	
None	217 (63.3)	79 (75.9)	

The use of insecticide-impregnated mosquito nets was the most known means of prevention in vector control (99.8%). The prevention of malaria in pregnant women by the combination of sulfadoxine and pyrimethamine was also known by most of the auxiliaries (77.4%). However, for 11.2% of the auxiliaries, quinine was reported to be an appropriate preventative measure (Table 3).

### Practices of auxiliaries

In the pharmacies visited, the auxiliaries advised patients regarding anti-malarial drugs. Approximately 91.3% of the auxiliaries had advised patients regarding anti-malarials at least once. Those with at least ten (10) years of service were the most likely (99%) to have provided this advice (Table 3). A statistically significant relationship between dispensing anti-malarials advice and having a certain number of years of service (p = 0.002) was observed. artemether + lumefantrine was the most recommended combination therapy (91.3%) for uncomplicated malaria, especially by untrained auxiliaries (97.1%) (Table 4).

Among the 408 auxiliaries who had ever recommended an anti-malarial drug, 71.1% reported correct dosages for patients over 14 years of age compared with those under 14 years of age (16.3%) (Table 5). A total of 60.3% of the auxiliaries who had not received specialized training recommended the correct doses of antimalarials compared with only 50.1% of those who had received training (Table 4).

**Table 5:** Recommended dosage according to the patient’s age

Age	Correct dosages		Incorrect dosages		*P value*
	Numbers (n)	Percentage (%)	Numbers (n)	Percentage (%)	
Patients ≤ 14 years old	199^a^	16.3	1025^a^	83.7	*0.0000001*
Patients over 14 years old	1450^a^	**71.1**	590^a^	28.9	
Total	1649	50.5	1615	49.5	

^a^Dosages were assessed by molecule

In this study, only 0.5% of the auxiliaries who dispensed anti-malarial drugs paired this with advice on malaria prevention. A total of 151 auxiliaries (33.8%) practicing in 52 private pharmacies performed RDT before recommending an anti-malarial drug, and only 28.6% of them, when faced with a negative test in a febrile patient, proposed symptomatic treatment and/or referred the patient to a health centre (Table 3).

## Discussion

The emergence of resistance to artemisinin derivatives in Southeast Asia poses a serious threat to other malaria-endemic areas. To delay this resistance, it is essential to apply the control measures recommended by the NMCP for correct management in private pharmacies. It is, therefore, important to assess the knowledge and practices of private pharmacy assistants, since they are the first point of contact. This information would be useful in guiding discussions on the involvement of the private sector in NMCP activities aimed at controlling or even eliminating malaria.

Inappropriate use of drugs remains a major public health problem worldwide [19, 20]. In developing and low-income countries, inappropriate drug use occurs due to the prescriptions and instructions given to clients on their use [6, 7]. Private pharmacies are an important link in promoting access to basic health services: they are the most accessible health facilities available for the management of common diseases [8]. The majority of families seek treatment for febrile illnesses at private pharmacies instead of at public health facilities [21, 22]. The first interlocutors are the auxiliaries. Thus, this study was interested in their level of knowledge and practices in private pharmacies in Abidjan regarding the management of malaria. The majority of these auxiliaries were the young with a secondary level of education. Niamkey and Yavo made the same observation in 2015 [23]. Pharmacists prefer to employ women because of their reception, attention, listening, and advice skills, which are essential to the smooth running of a pharmacy. In addition, because of certain difficulties challenging the auxiliary profession, such as standing all the time and the long working hours, which are difficult for older people, young people are preferred. In most cases, these are young people who have dropped out of school and have chosen to become auxiliaries, regardless of having received specialized training.

The malaria knowledge of the auxiliaries varied from one question to another. Knowledge of clinical signs, biological tests, antimalarial drugs, vector control, and intermittent preventive treatment with sulfadoxine–pyrimethamine (IPT + SP) in pregnant women was good. However, the pathogen and the national guidelines for malaria management were not well-known by the auxiliaries. Some clinical manifestations specific to malaria constitute benchmarks for the auxiliaries to propose anti-malarial drugs for curative treatment. These include fever, headache, asthenia, and body aches. These signs have also been described by other authors with varying frequency [24–26]. However, because these signs are not uniquely linked to malaria, their use as the only criteria for diagnosing the disease would lead to the abusive use of antimalarial drugs [18] and, therefore, to the selection of resistant strains of *Plasmodium* to anti-malarial drugs. Therefore, the use of RDTs for malaria must be popularized in pharmacies by providing them out at a lower cost or even free of charge. As such, the treatment of confirmed cases of malaria only will contribute to a more effective fight against *Plasmodium* through effective and correct management of this disease. Currently, some rapid diagnostic tests have sensitivity comparable to that of routine microscopy, so they can be used by paramedics to confirm malaria cases encountered in the pharmacy [27]. However, the biological confirmation of malaria is not performed in all pharmacies before dispensing anti-malarial drugs. In our study, RDTs were not frequently performed prior to dispensing an anti-malarial drug. Artemisinin-based combination therapy (ACT) was better known by the auxiliaries.

Our results are similar to those of Niamkey and Yavo [23], who found that 91.3% and 78.3% of auxiliaries knew of artemether + lumefantrine and artesunate + amodiaquine, respectively. In general, the anti-malarials were known to the auxiliaries. This could be explained by the presence of many types of these artemisinin-based combinations in the pharmacies due to commercial competition. Thus, the therapeutic choice of these artemisinin-based combinations could be influenced by the medical sales representatives who visit the pharmacies, or by price differences. In Angora’s study in Abidjan, artemisinin derivatives was the anti-malarial drug most known, by 48.4% of the auxiliaries [18]. Although the pharmacy auxiliaries were aware of ACT, only approximately 45.9% of them were aware of the existence of a NMCP and its guidelines. In Côte d’Ivoire, the NMCP only recommends the use of AL or AS + AQ or dihydroartemisinin and piperaquine as first-line treatment and oral quinine as second-line treatment [5]. However, these results showed that the majority were not familiar with the national guidelines for the management of uncomplicated or severe malaria. Elsewhere on the continent, the NMCP is better known. For example, Ganfon et al. reported, from their study conducted in private pharmacies in five major cities in Benin, Burkina Faso and Mali, that 84% of respondents were aware of the NMCP [9]. In addition, 38.3% of these individuals were aware of their country’s national malaria control protocol, and 10.6% had the official document [9]. The results of these study (knowledge of PNLP, sources of information) showed that the NMCPs have performed few activities aimed at pharmacy assistants in recent years, as the main sources of information for assistants on malaria were visits by medical representatives and training organized by laboratories. The NMCP should involve private pharmacies because approximately 40% of their clients attend for suspected malaria [28, 29]. As for the pharmacists, they represented only 23%; and once every 2 weeks, the auxiliaries benefited from training organized by the attending pharmacist. Therefore, continuous training must be regularly provided to these assistants. Moreover, the auxiliaries were also found to have in-depth knowledge of vector control methods. Several awareness campaigns have been conducted for several years by the Ministry of Health of Public Hygiene and Universal Health Coverage (MSHP-CMU) to achieve 100% coverage and 80% use of LLITNs in the general population [2]. In addition, sulfadoxine + pyrimethamine was the most-cited preventive drug for pregnant women; and some of them stated that a vaccine that protects against malaria is available. This could be explained by confusion between vaccines and certain injectable forms of anti-malarial drugs, because most vaccines are administered by injection. The proportion of those with the notion of the existence of a vaccine against malaria, although present in this study, has decreased in comparison with that reported in surveys conducted a few years earlier [30]. These results reflect the dissemination of information on malaria prevention by the MSHP-CMU. The present findings showed that auxiliaries not trained at the auxiliary training institutions were better at answering questions. The high proportion of correct answers provided by untrained auxiliaries was due to the existence of other sources of information (medical sales representatives, laboratories, media), the filling of medical prescriptions, and experience in the profession. In addition, working for several years provides professional experience and knowledge, which enable the acquisition of skills and the execution of appropriate practices. The majority of the auxiliaries recommended anti-malarial drugs to the patients. The combination of artemether + lumefantrine was the most recommended for the curative treatment of simple malaria. However, the administration of these drugs was not followed by advice on prevention tools, which is the only method of improving malaria control and reducing the incidence of the disease [31].

The combination artesunate + amodiaquine, although known, was the least recommended in our study. According to the auxiliaries, this combination is poorly tolerated by the patients. Thus, when faced with a case of malaria, most of the auxiliaries preferred anti-malarial drugs without amodiaquine.

Sulfadoxine + pyrimethamine (SP) was recommended by the auxiliaries for the curative treatment of uncomplicated malaria and for intermittent preventive treatment in pregnant women. This finding confirms that auxiliaries do not apply NMCP guidelines. According to NMCP guidelines, SP is only used in pregnant women as an intermittent preventive treatment [2, 5].

Auxiliaries with at least 10 years of experience were the most likely to recommend anti-malarials, especially the combination of artemether + lumefantrine. These anti-malarial drugs were recommended at the correct dosage for patients over 14 years of age, in contrast with for those under 14 years of age. The dosage of anti-malarials in patients under 14 years of age in most cases is calculated according to body weight, which is burdensome for auxiliaries with less experience.

The dispensing and delivery of drugs to patients in private pharmacies are activities for which the pharmacist is directly responsible. The pharmacist has a special duty to advise patients when dispensing drugs that do not require a medical prescription [11]; hence, their daily presence with the assistants who help them with these tasks is important.

The current study had limitations in that it was a one-off survey and, therefore, could not assess the impact of other factors on auxiliary knowledge and practices. Furthermore, the current results are based on a sample of 163/486 pharmacies in Abidjan. It would be essential to carry out a larger survey in a wider coverage of pharmacies both in Abidjan and in other cities of Ivory Coast, in order to appreciate the knowledge and practices of auxiliaries. Not all the auxiliaries who were approached gave answers, and they might have had different characteristics from those who took part in this study. However, despite these limitations, important information was obtained on the knowledge and practices of the auxiliaries. This study could help the NMCP to better orient malaria management strategies.

## Conclusion

The epidemiology of malaria and its management, as well as the NMCP recommendations for the diagnostic and therapeutic management of malaria, are not well known to auxiliaries, which may have implications for their practices. The overuse of anti-malarial drugs at incorrect doses can lead to the selection of resistant strains or toxicity. Achieving a substantial reduction in malaria cases in Africa will be challenging. In principle, these modules are taught in training schools, hence the need to monitor the content of the modules delivered by these schools, as well as the qualifications of the teachers. It seems obvious that regular refresher courses are needed to correct the deficiencies observed and ensure that these auxiliaries have a good level of knowledge and the right attitudes. To achieve the aim of zero malaria and eradicating malaria by 2030, private sector involvement will be necessary. Thorough knowledge of national malaria epidemiology and guidelines in private pharmacies will allow for proper basic therapeutic management and help prevent *Plasmodium falciparum* from rapidly developing ACT resistance.

## Data Availability

The datasets used and/or analysed during the current study are available from the corresponding author on reasonable request (email: akouaval@yahoo.fr).

## Abbreviations

WHO: World Health Organization
ACT: Artemisinin-based combination therapy
AL: Artemether + lumefantrine
AS + AQ: Artesunate + amodiaquine
DHAP: Dihydroartemisinin + piperaquine
ASPY: Artesunate + pyronaridine
SNPP-CI: National Union of Private Pharmacists of Côte d'Ivoire (Ivory Coast)
SPSS: Statistical Package for Social Science
RDT: Rapid Diagnostic Test
NMCP: National Malaria Control Programme
IPT + SP: Intermittent Preventive Treatment with Sulfadoxine–Pyrimethamine
